# Identifying national health research priorities in Timor-Leste through a scoping review of existing health data

**DOI:** 10.1186/1478-4505-11-8

**Published:** 2013-03-01

**Authors:** Jacqueline Deen, Livio da Conceicao Matos, Beth Temple, Jiunn-Yih Su, Joao da Silva, Selma Liberato, Valente da Silva, Ana Isabel Soares, Vijaya Joshi, Sarah Moon, James Tulloch, Joao Martins, Kim Mulholland

**Affiliations:** 1 Menzies School of Health Research, Royal Darwin Hospital Campus, Rocklands Drive, Casuarina NT 0811, Australia; 2 Ministry of Health, Caicoli, Dili, Timor-Leste; 3 Charles Darwin University, Darwin, NT 0909, Australia; 4 AusAid, 255 London Circuit, Canberra, ACT 2601, Australia; 5 Universidade Nacional de Timor Lorosa’e, Avenida Cidade de Lisboa, Díli, Timor-Leste; 6 London School of Hygiene and Tropical Medicine, Keppel Street, London WC1E 7HT, UK

**Keywords:** Timor-Leste, East Timor, Health management information system, Health research

## Abstract

Health research is crucial to understand a country’s needs and to improve health outcomes. We conducted a scoping review and analysis of existing health data in Timor-Leste to identify the health research priorities of the country. Published and unpublished health research in Timor-Leste from 2001 to 2011 that reported objectives, methods and results were identified. Key findings were triangulated with data from national surveys and the Health Management Information System; 114 eligible articles were included in the analysis, the leading topics of which were communicable (malaria, tuberculosis, HIV and sexually transmitted diseases and dengue) and non-communicable (eye and mental health) diseases. There were 28 papers (25%) on safe motherhood, child health and nutrition, of which 20 (71%) were unpublished. The review of national indicators showed high infant, under-five and maternal mortality rates. Burden of disease is greatest in young children, with respiratory infections, febrile illnesses and diarrheal disease predominating. There is poor access to and utilization of health care. Childhood malnutrition is an important unresolved national health issue. There are several obstacles leading to under-utilization of health services. The following topics for future health research are suggested from the review: nutrition, safe motherhood, childhood illness (in particular identifying the causes and cause-specific burden of severe respiratory, febrile and diarrheal diseases) and access to and use of health services.

## Background

Health research is essential for developing rational health policies and services [1]. Developing country governments are increasingly recognizing the value of research as an investment for better health. The Democratic Republic of Timor-Leste commonly known as Timor-Leste or East Timor, is a small country in Southeast Asia. Its health infrastructure was completely devastated during the violence that followed the September 1999 referendum vote to separate from Indonesia. Following independence in May 2002, the new government, assisted by a United Nations mission, is rehabilitating its health system. Although several strategies and policies have been implemented in Timor-Leste during the last decade, they have not always been guided by evidence-based research and analysis. In the last 10 years, clinical researchers and non-governmental organizations (NGO) have undertaken studies in Timor-Leste. However, Timor-Leste is one of the countries with the fewest publications in medicine, both in absolute numbers and per capita [2]. In 2010 the Timor-Leste Cabinet for Health Research and Development (CHR&D) was established to facilitate and promote health research [3].

We conducted a scoping review and analysis of existing data in Timor-Leste to assess the health-related studies that have been conducted in the country, identify gaps and inform the development of priority areas for future health research. This assessment of existing health data consisted of a literature review and analysis of key health indicators from several sources.

## Methods

### Study site and population

Timor-Leste is divided into thirteen districts [4] with the capital, Dili, located in the district of the same name. The total population in 2010 was 1,066,409 with a high annual growth rate of 2.4% [5]. The population is young with 41.4% less than 15 years of age. At present, Timor-Leste has the following health facilities: 67 community health centers (some with beds for in-patients), 114 health posts, Community Health Integrated Service mobile clinics, five district hospitals and one national referral hospital (National Hospital Guido Valadares).

### Literature review

Studies from 2001 to 2011 on health and health-related issues in Timor-Leste were identified. Published studies were located in Pubmed and EBSCO databases using the search string “health” or “social” or “economic” AND “Timor” in the title and abstract fields. Unpublished studies, including theses and reports, were obtained from NGO and international organization compilations, a CHR&D collection of research documents and through author contacts. The published and unpublished articles were compiled in Endnote (Thomson Reuters, San Francisco, CA, USA). Titles and abstracts were screened for eligibility. Articles reporting objectives, methods and results were included, whereas policy and situational analyses, trip narratives, strategies, proposals, plans, fact sheets, appeals, program updates, promotional materials, news updates, editorials, opinion pieces, viewpoints and case descriptions were excluded. Incomplete documents and studies that described expatriates living in Timor-Leste or Timorese permanently residing outside the country were also excluded. The relevant full papers were downloaded and reviewed in detail. Information from each eligible paper was extracted and entered into an Excel spread sheet (Microsoft Office 2007, Seattle, WA, USA) and each article was classified according to the primary health care key indicators of Timor-Leste [6], as follows: safe motherhood, family planning and reproductive health, child health, nutrition and food security, communicable diseases, health promotion, environmental health, vector control, non-communicable diseases, pharmacy, provision of and access to health services, and other topics.

The data were analyzed in aggregate to obtain a landscape picture of the number of studies by year and by topic. The studies were classified as qualitative (defined as studies that aim to understand human behavior and the reasons for such behavior) or quantitative (defined as research that classify characteristics and count them, with or without an intervention). Key results and important gap areas were identified. Towards the goal of comprehensiveness, all studies that met the basic standard requirements were included and articles were not excluded based on quality of methodology or reporting.

### Analysis of key health indicators

Data were obtained from the 1997 Indonesia Data Demographic and Health Surveys (DHS) [7], the 2002 Multiple Indicator Cluster Survey (MICS) [8], the 2003 Timor-Leste DHS [9], the 2007 Timor-Leste Survey of Living Standards (SLS) [10], the 2009–10 Timor-Leste DHS [11] and the World Health Organization (WHO) 2011 World Health Statistics [12]. Information was also included from the Timor-Leste Health Management Information System (HMIS), the office that collates monthly paper-based reports from community health centers, health posts, mobile clinics, private clinics, district hospitals and the national referral hospital on mainly clinically-diagnosed, high priority diseases.

Child (infant and under-five year) and maternal mortality rates, causes of morbidity and health service indicators across the continuum of care were assessed. The health service indicators included family planning, perinatal care, childhood immunization coverage and nutritional status, as well as access to and utilization of health services (Table 1). Trends in national level indices were evaluated, while acknowledging that the sample size, design, study population, period of coverage, and methods of estimation were not standardized across these reports. Since Timor-Leste is classified as a lower middle-income country [13], its key indicators were compared with those of neighboring lower middle-income countries (Indonesia, Papua New Guinea, Philippines and Vietnam) using data from the WHO World Health Statistics [12].

**Table 1:** Description of health indicators across the continuum of care

**Health area**		**Indicators and definition**
Mortality	Infant mortality rate	Probability of dying between birth and one year of age, per 1,000 live births
	Under-five mortality rate	Probability of dying between birth and five years of age, per 1,000 live births
	Maternal mortality rate	Annual number of maternal deaths per 1,000 women aged 15 to 49 years
	Maternal mortality ratio	Age-standardized maternal mortality rate divided by age-standardized general fertility rate, per 100,000 live births. It is often considered the more useful measure of maternal mortality as it measures the obstetric risk associated with each live birth.
Causes of morbidity	Incidence of top 10 notifiable diseases	Annual number of cases presenting for treatment to health facilities divided by the projected population for that year, by age group
Family planning	Total fertility rate for women aged 15 to 49 years	Total estimated number of births a woman would have by the end of her childbearing period
	Contraceptive use	% of married women 15 to 49 years currently using any method of contraception
Perinatal care	Antenatal care	% of mothers who had at least four antenatal care visits
		% of mothers who received antenatal care from skilled health personnel
	Skilled birth attendance	% of births delivered by skilled health personnel
	Place of delivery	% of births delivered in a health facility
	Birth weight	% of births with reported birth weight
	Low birth weight	% of births less than 2.5 kg
Immunization	DPT coverage	% children who received 3 doses of DPT (HMIS data is for children < one year of age, whereas for all other sources it is for children 12 to 23 months)
	Full immunization	% children 12 to 23 months who are fully immunized (received BCG, measles and 3 doses of DPT and polio vaccines)
Nutrition	Breastfeeding	% of children under six months old who were breastfed six or more times in the 24 hours preceding the interview
		% children up to five months old exclusively breastfeeding
	Complementary feeding	% children six to nine months old receiving complementary foods
	Anthropometric indices*- % children under five years old with:	Moderate stunting - height-for-age z-score below −2 standard deviations (SD)
		Severe stunting - height-for-age z-score below −3 SD
		Moderate wasting - weight-for-height z-score below −2 SD
		Severe wasting - weight-for-height z-score below −3 SD
		Moderate undernutrition - weight-for-age z-score below −2 SD
		Severe undernutrition - weight-for-age z-score below −3 SD
	Anemia	% children under five years old with hemoglobin <110 g/dL

*Anthropometric indices: Stunting reflects the cumulative and chronic effects of malnutrition and infection starting *in-utero* and has the most serious and long-lasting health impact. Wasting indicates acute weight loss. Although undernutrition is a composite indicator that may reflect stunting or wasting and be difficult to interpret, it may be more accurate as it does not include height in its calculation. The anthropometric indices were based on the former NCHS/CDC/WHO international reference values in the MICS and the 2003 DHS, and on the latest WHO Child Growth Standards in the SLS, 2009–10 DHS and WHO World Health Statistics.

### Ethics

This study involved the collection and analysis of freely available de-identified existing data, documents, and records. The study received ethical approval from the Technical and Ethical Committee of the Cabinet of Health Research and Development of Timor-Leste.

## Results

### Selection of articles

**Figure 1:**
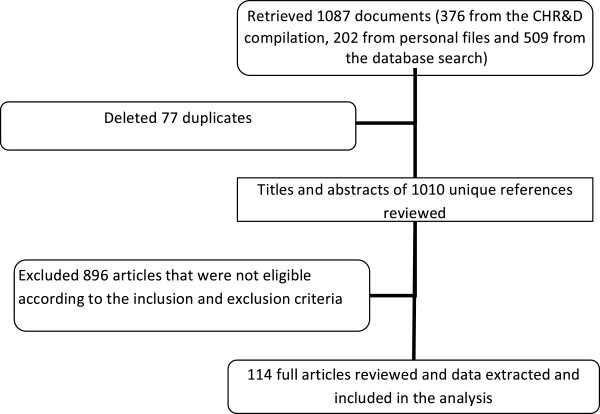
Selection of articles for the existing health data review in Timor-Leste, 2001 to 2011.

**Figure 2:**
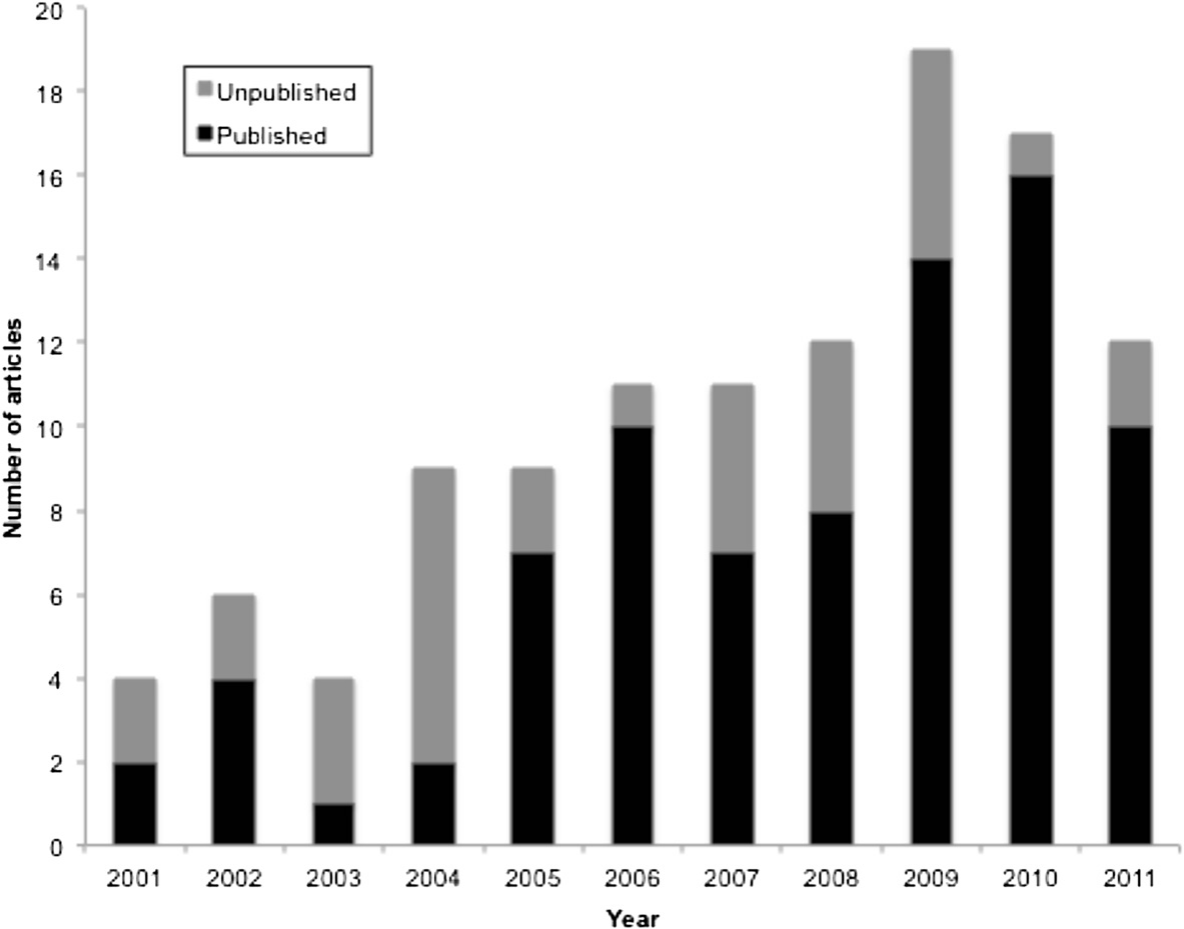
Health research papers from Timor-Leste, 2001 to 2011, by year.

**Figure 3:**
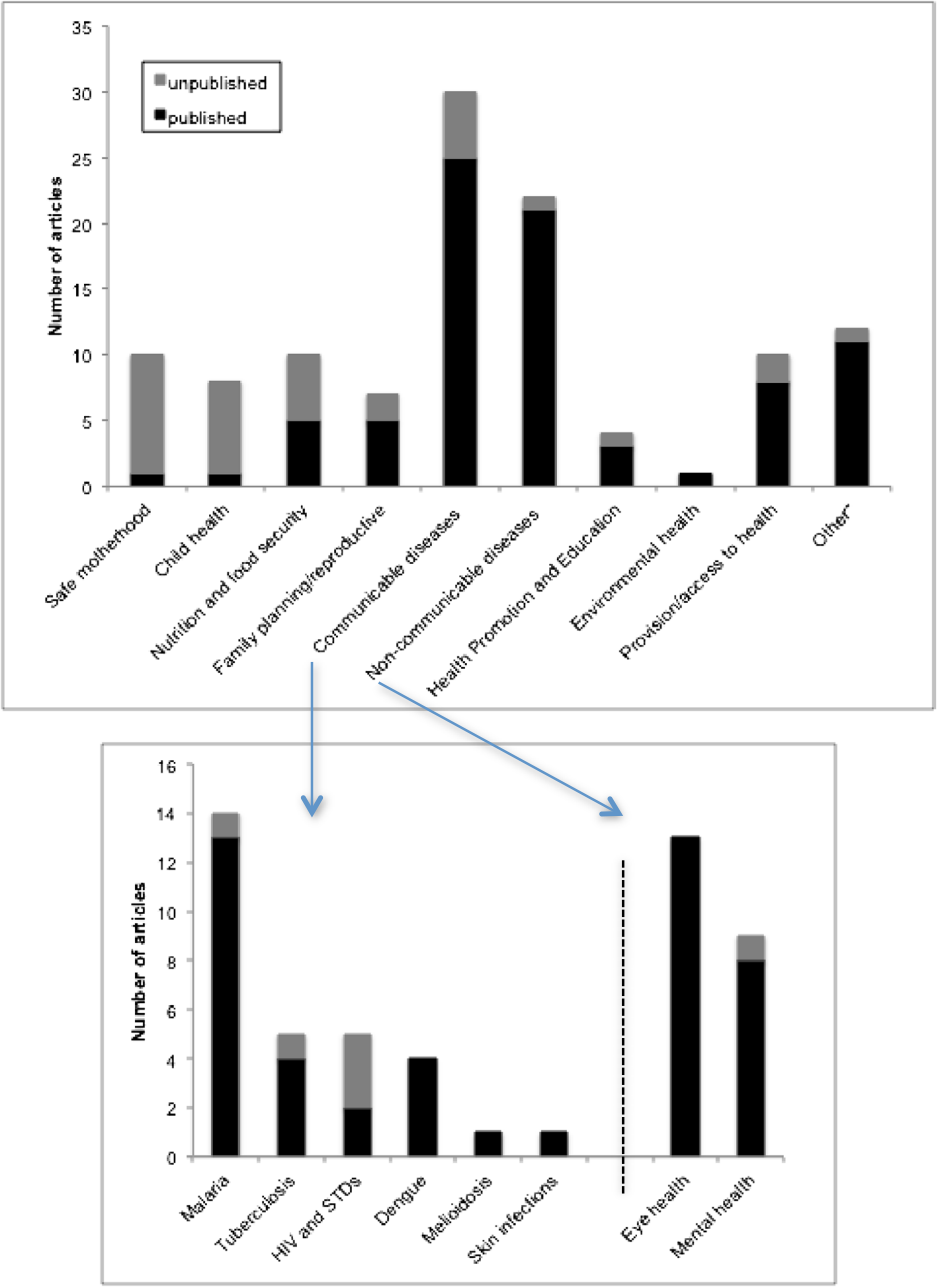
Health research papers from Timor-Leste, 2001 to 2011, by topic.

A total of 1,087 articles were retrieved (Figure 1). After deleting duplicates, the titles and abstracts of 1,010 unique references were reviewed. There were 114 eligible articles, 81 (71%) published and 33 (29%) unpublished, included in the analysis [List of 114 references in Additional file 1]. The number of papers by year of report or publication is shown in Figure 2. Of the 114 papers the leading topics were communicable (30; 26%) and non-communicable (22; 19%) diseases, of which 46/52 (88%) were published (Figure 3). Forty seven percent (14/30) of communicable disease articles were on malaria. The non-communicable disease subjects comprised eye (13/22; 59%) and mental (9/22; 41%) health. There were 28/114 (25%) articles on safe motherhood, child health and nutrition, of which 21 (75%) were unpublished. Of the 114 articles, 42 (37%) were qualitative studies, 59 (52%) had a quantitative design and 13 (11%) were combined qualitative and quantitative research.

### Child and maternal mortality

**Figure 4:**
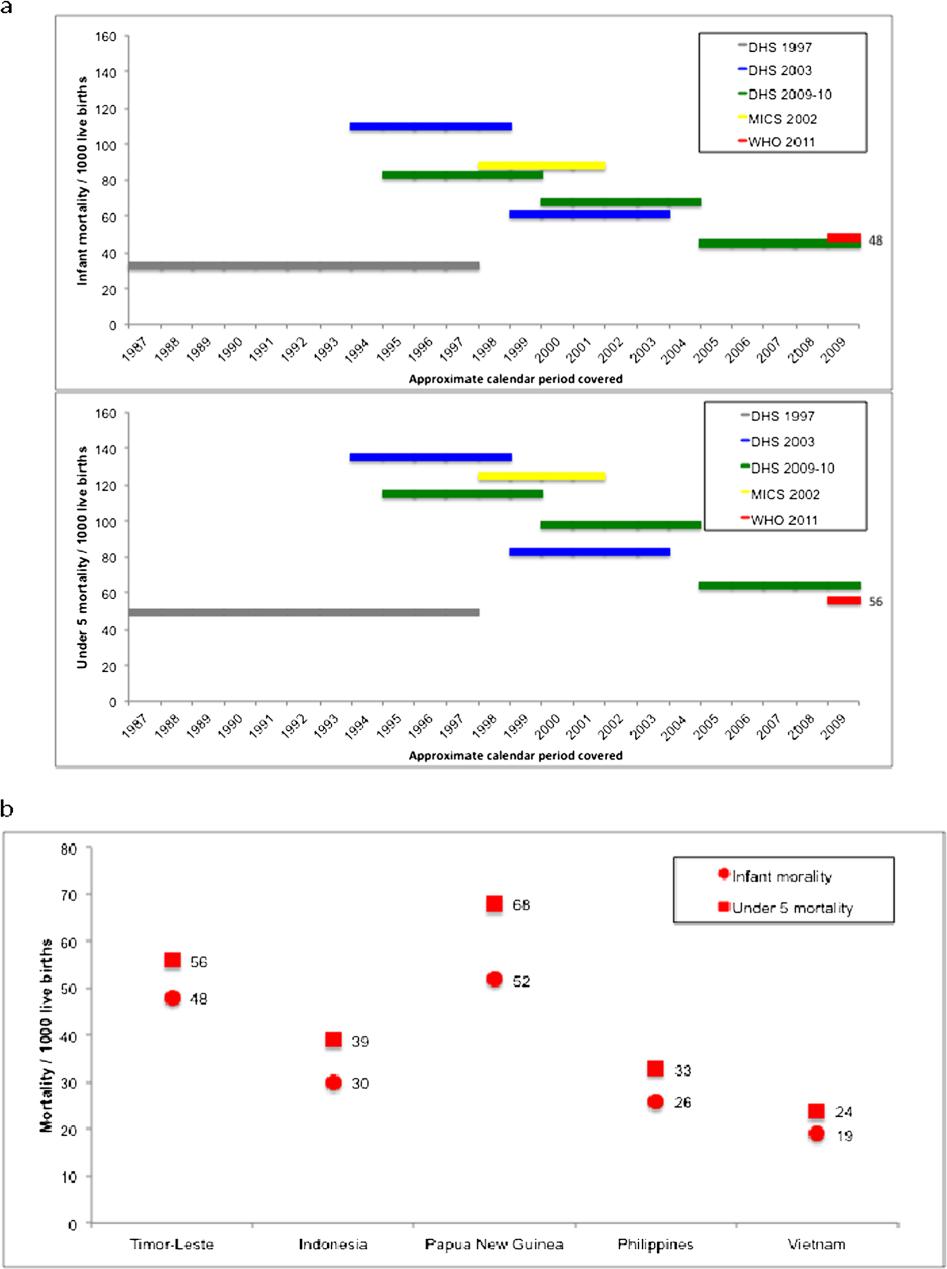
**Estimates of infant and under-five-year old mortality.** (**a**) For Timor-Leste, by approximate calendar period covered and data source [7-9,11,12]; (**b**) For Timor-Leste and neighboring lower middle-income countries in 2009 from the WHO World Health Statistics 2011 [12].

The three DHS surveys [7,9,11], the MICS [8] and the WHO World Health Statistics [12] included estimates of infant and under-five mortality rates in Timor-Leste, covering the period 1987 to 2009 (Figure 4a). The approximate calendar period covered by the surveys overlap since child mortality estimates were based on reports of survivorship of children by their mothers. Excluding the 1997 Indonesia DHS survey [7], estimates show a steady decline in both infant and under-five mortality rates (Figure 4a). The latest figures from the WHO World Health Statistics [12] estimate the infant and under-five mortality rates at 48 and 56 per 1,000 live births, respectively. These rates are relatively high compared with neighboring middle lower-income countries, although lower than those of Papua New Guinea (Figure 4b).

**Figure 5:**
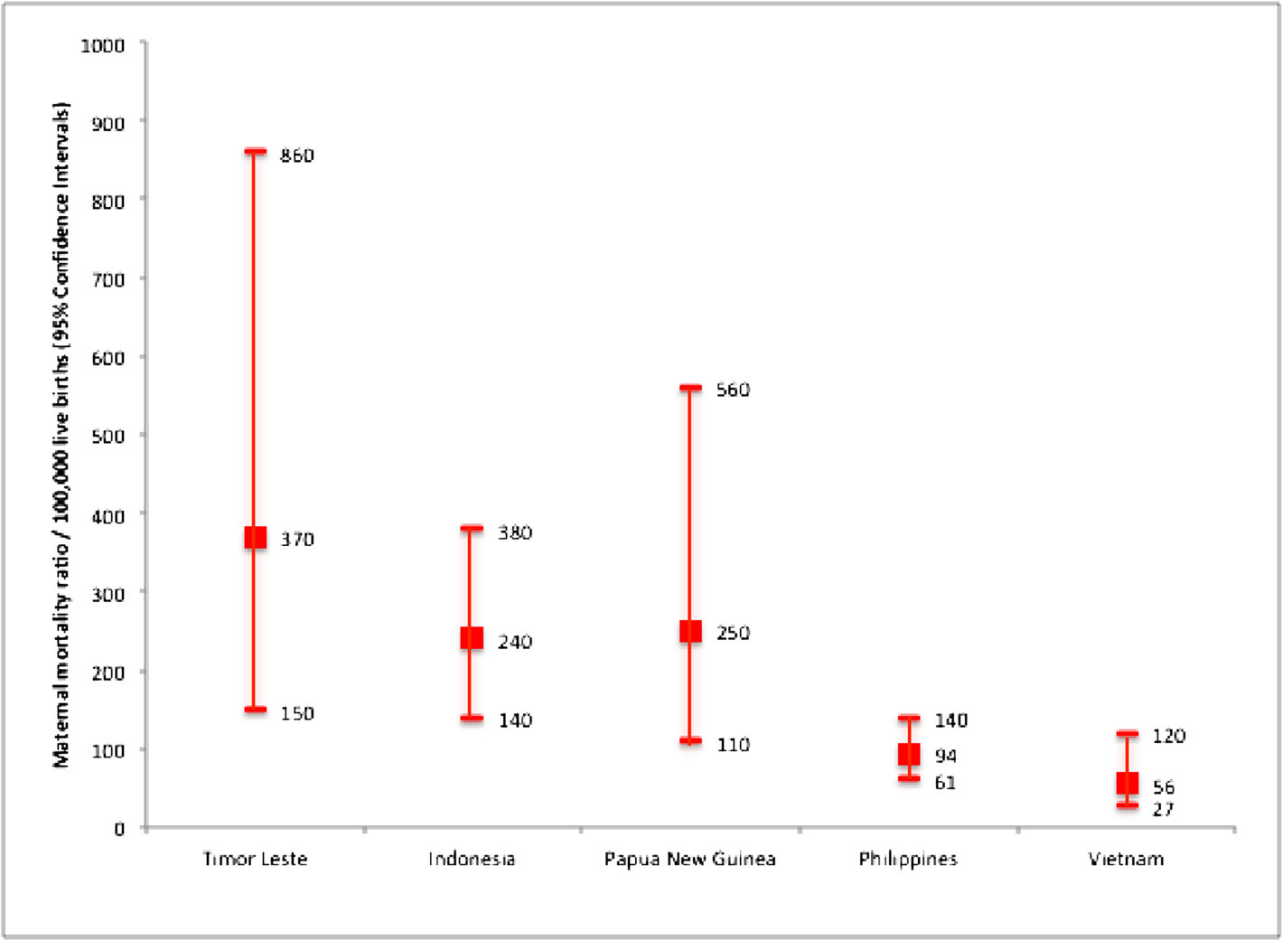
**Estimates of maternal mortality ratio/100,000 live births for Timor-Leste and neighboring lower middle-income countries in 2008 from the WHO World Health Statistics 2011**[12]**.**

The 2009–10 DHS [11], which provides the first direct maternal mortality estimates in Timor-Leste from a population-based survey, found a maternal mortality rate of 0.96 per 1,000 woman-years and a maternal mortality ratio of 557 (95% CI: 408 to 706) per 100,000 live births for the period 2003 to 2009. The WHO World Health Statistics has a lower estimate for maternal mortality ratio for Timor-Leste in 2008 (370; 95% CI: 150 to 860); however, this figure was still higher than those for neighboring middle lower-income countries (Figure 5).

Key findings from the literature on perinatal mortality were examined. In 2009, Health Alliance International (2009) conducted a qualitative study in six districts examining neonatal deaths [Health Alliance International, unpublished data]. Midwives from community health centers and health posts were interviewed on events surrounding 72 known perinatal or neonatal deaths within their catchment areas. Fifty-three (74%) of the reported deaths were of babies delivered at home. Of the 54 live births, 42 (78%) died within the first 24 hours, of which 41 (98%) died within the first six hours of life.

### Causes of morbidity and mortality

**Figure 6:**
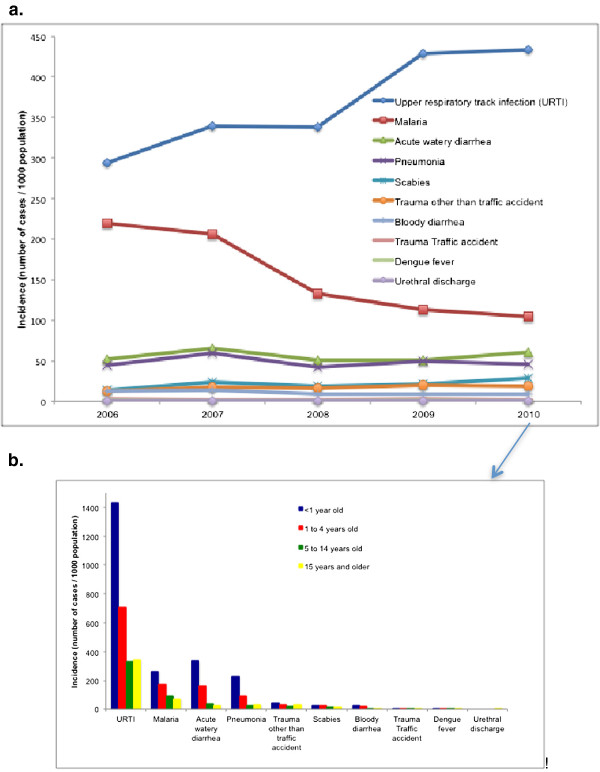
**Top ten notifiable diseases, Timor-Leste Health Management Information System.** (**a**) For all ages, by year (2006 to 2010); (**b**) In 2010, by age group.

Of the 28 notifiable diseases reported through HMIS, the top ten are consistent from 2006 to 2010, with upper respiratory tract infection the most common every year, followed by malaria (Figure 6a). The top notifiable diseases correlate very closely with the findings from a large health care seeking behavior study of 25,000 individuals in Timor-Leste in 2009 [14]. In that study 5,818 (23%) people reported that they had experienced a health problem in the previous 30 days. The most common complaints were cough (32%), fever (14%) and malaria (12%).

Categorization by age group of the top notifiable diseases reported through HMIS in 2010 shows that the burden of disease is highest among children (Figure 6b). From the literature, acute respiratory infections, febrile illnesses and diarrhea also stand out as the most common cause of illness and death in children. For example, interviews of 800 women, 15 to 44 years of age in 2002 in Aileu district, found that the most commonly reported causes of childhood death were probable pneumonia, tuberculosis or whooping cough (described as a strong cough with fever and weight loss); probable malaria (described as a high fever and sometimes an accompanying seizure); dehydration and shock caused by dysentery or diarrhea; measles and malnutrition [Livermore C, unpublished data]. A retrospective study [Bucens I and Barreto A, unpublished data] found that the most common diagnosis on admission of children to the national referral hospital are respiratory tract infection (44%; mainly pneumonia or bronchiolitis) diarrheal disease (17%), febrile illness due to malaria, dengue or central nervous system infection (13%), tuberculosis (9%) and malnutrition (5%). In that study the most common causes of death were respiratory tract infection (29%), central nervous system infection (16%) and diarrheal disease (14%).

### Indicators of health service coverage and continuum of care

Key health indicators across the continuum of care from different data sources were collated and compared (Table 2). The period immediately following the 1997 Indonesia DHS Survey and the 1999 referendum vote, characterized by violence and upheaval, showed the worst markers. However, there are definite indications of improvement during the past decade for family planning, perinatal care and immunization coverage.

**Table 2:** **National key health indicators, by data source **[8-11]

	**DHS 1997**	**MICS 2002**	**DHS 2003**	**SLS 2007**	**HMIS**				**DHS 2009/10**	**WHO**
**Family planning - calendar period covered, unless indicated**	**1995 to 1997**	**2001**	**2001 to 2003**						**2007 to 2009**	**2009**
Total fertility rate for women aged 15 to 49 years	4	8	8						6	6
% married women 15 to 49 years of age currently using any method of contraception	27 (1997)	8 (2002)	10 (2003)	20 (2007)					22 (2009 to 2010)	22 (2000 to 2010)
**Perinatal care - calendar period covered, unless indicated**	**1993 to 1997**	**2001**	**1999 to 2003**	**2007**	**2007**	**2008**	**2009**	**2010**	**2005 to 2009**	**2000 to 2010**
% of mothers with at least four antenatal care visits (% with any antenatal care from skilled health personnel)	(70)	(43)	(61)		31	35	45	42	55 (86)	55
% live births delivered by skilled health personnel	26	24	19	41	35	36	46	49	30	30
% births delivered in a health facility	16		10						22	
% low birth weight deliveries (% of births with reported weight)	6 (20)	8 (10)							10 (26)	12 (2000 to 2009)
**Immunization - calendar period covered, unless indicated**	**1997**	**2001**	**2003**	**2007**	**2007**	**2008**	**2009**	**2010**	**2009 to 2010**	
% children who received three doses DPT	63	17	38	76	70	79	73	72	66	
% children 12 to 23 months fully immunized	56	5	18	27					46	
**Nutrition - calendar period covered, unless indicated**	**1997**	**2001**	**2003**						**2009 to 2010**	
% children under six months of age who were breastfed six or more times in the 24 hours preceding the interview	95								98	
% children up to five months old exclusively breastfeeding	31	44	31						52	
% children six to nine months old receiving complimentary foods		63	82						80	
% children six to 59 months old with any anemia			32						38	

Total fertility rate for women aged 15 to 45 years decreased from eight to six births and the percentage of mothers who received skilled antenatal care increased from 43% to 86% (Table 2). However, the percentages of skilled birth attendance and health facility deliveries still remain low and may be an important factor in maternal mortality. The literature review identified some research into skilled birth attendance and place of delivery. Harrison [15] examined the factors associated with skilled birth attendance in six districts. In a cross-sectional sample of 301 women aged 15 to 49 who had given birth in the previous two years, only 32% had a doctor, nurse or midwife present at birth, and 15% received postpartum care within three days of delivery. Women’s educational level, parity, household wealth index, and having received four or more antenatal care visits were significantly associated with skilled birth attendance. Van Schoor [unpublished data] interviewed 120 respondents (parents, traditional birth attendants, midwives and community leaders) in Viqueque district and found that prior experience and confidence in the skills of traditional birth attendants are a main factor in deciding to deliver at home, although physical access especially for night time deliveries prevent some women from getting to health facilities.

Childhood vaccine coverage rates have improved dramatically over the past decade, but still remain relatively low with DPT-three coverage estimated at 66% to 72% in recent years (Table 2). Although 98% of children less than six months were breastfed six or more times in the day prior to the 2009/10 DHS interview, only 52% were exclusively breastfed. Nearly 40% of Timorese children six to 59 months of age are anemic.

**Figure 7:**
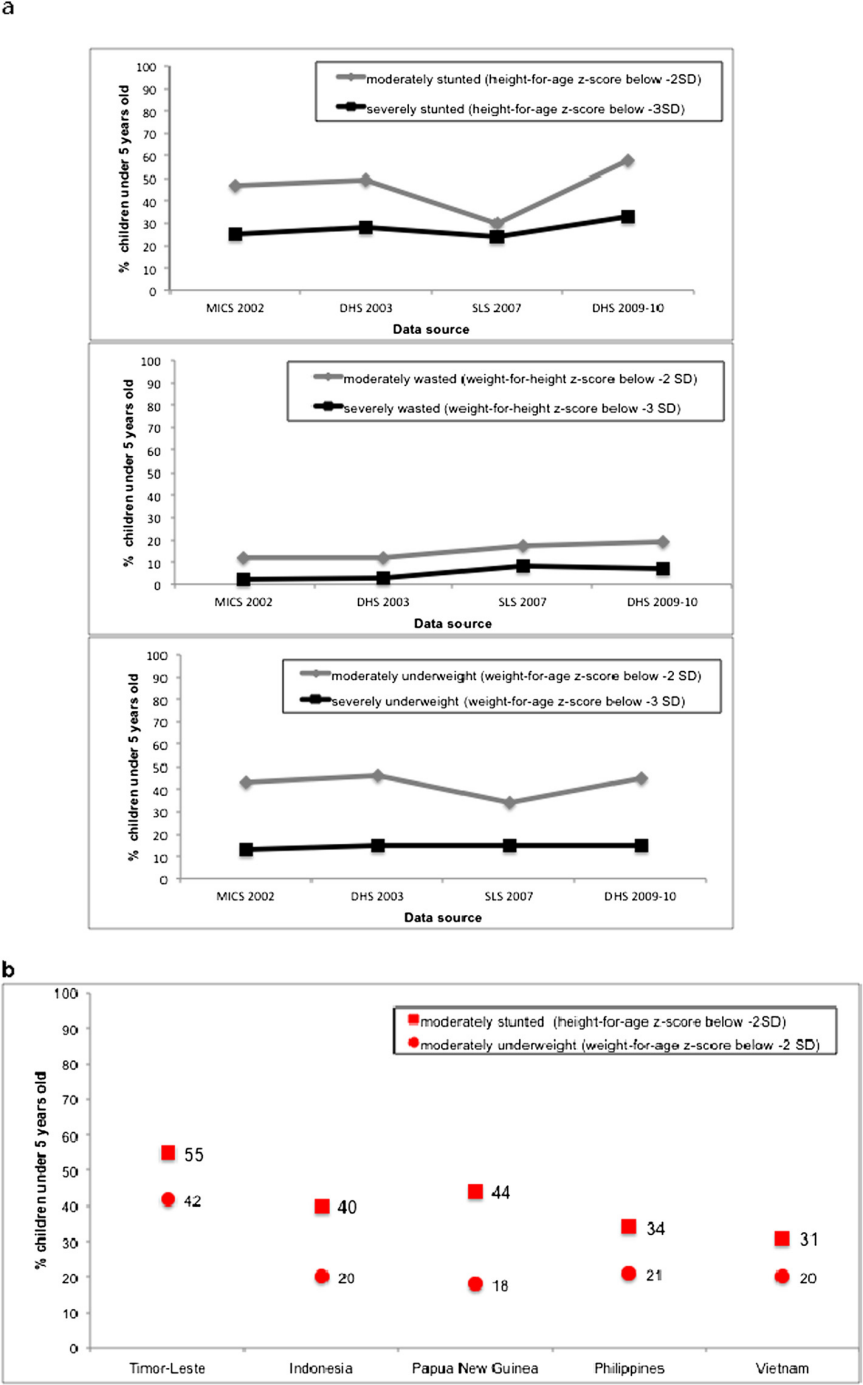
**Anthropometric indices of children under five years of age.** (**a**) For Timor-Leste, by data source [8-11]; (**b**) For Timor-Leste and neighboring lower middle-income countries between 2000 to 2009 from the WHO World Health Statistics 2011 [12].

There has been little improvement in childhood malnutrition during the last decade with moderate stunting in over half of children under five years of age (Figure 7a). Based on the WHO estimates [12] anthropometric indices in Timor-Leste indicate 55% stunting and 42% undernutrition of children under five, which are worse than those of neighboring low middle-income countries (Figure 7b). These national estimates are corroborated by information from the literature review. A 2003 survey in Baucau and Viqueque [Bau AM and Melito C for the Government of Timor-Leste and German GTZ, unpublished data] that included 846 children six to 59 months of age and their mothers showed that 54% and 19% of the children had moderate stunting and moderate wasting, while 25% of the mothers had a body mass index below 18.5 kg/m^2^. A 2004 evaluation of 900 children six to 59 months of age in the low- and high-lands of Liquica, Covalima and Bonobaro found 53% and 59% moderate stunting and 15% and 13% moderate wasting, in the low- and highlands, respectively [Dubray C and Rose AMC for Epicentre and Care International Timor-Leste, unpublished data]. Considering the sampling differences and likely inter-observer variability across the national and district surveys, the similarities in the stunting and wasting rates are striking. From 2000 to 2003, 280 (33%) of 880 children admitted to the national referral hospital were malnourished, of whom 61% were severely wasted and 54% were both wasted and stunted with a hospital case-fatality rate of 36/280 or 12.9% [16].

Various surveys assessed access to and utilization of health services. According to the 2003 DHS [9], 12% of households reported not using any health care provider when a household member was ill and 25% of households were 2 or more hours away from the usual first health care provider. Curative health care encounters occurred predominantly in community health centers (59%), followed by government hospitals (25%) and private clinics (11%). The 2007 SLS [10] found that the average distance from a household to a treatment centre was 2.8 km (3 and 2 km in rural and urban areas, respectively) with a travel time of 54 minutes one-way (60 and 34 minutes in rural and urban areas, respectively). In the 2009–10 DHS [11], 96% of women age 15 to 49 reported that they have at least one problem in accessing health care for themselves. They identified the following difficulties in accessing health care: non-availability of drugs in the treatment centre (87%), absence of a health care provider (82%) or a female provider (63%); having to take transport (59%); concern about distance to the health facility (53%); not wanting to go alone (43%) and having to get permission (23%). In the 2009 health care seeking behavior study in Timor-Leste [14], travel time to a health facility for the most recent visit by a household member was less than 1 hour for 45%, over two hours for 29% and over five hours for 5%. A qualitative study in five sub-districts found that the major barriers to health care utilization were socio-behavioral and service quality issues, geographical distance, as well as interchangeable use of biomedical and traditional health services [Edmonds A for the World Bank, unpublished data].

## Discussion

Nutrition, maternal health, childhood morbidity and mortality (in particular identifying the causes and cause-specific burden of cough illnesses such as pneumonia and tuberculosis, febrile illnesses such as malaria, dengue and tuberculosis, and diarrheal diseases) and access to and use of health services are proposed as national priority areas for research. The purpose of the review is to make recommendations about research and not about priority interventions such as those included in the basic services package for primary health care and hospitals [17]. Our analysis was structured around the key health indicators of Timor-Leste and provides a broad-brush picture of health issues where more information is needed.

Childhood malnutrition continues to be a major challenge in Timor-Leste. Our findings are corroborated by a recent assessment of global trends in undernutrition [18]. The analysis found that although children’s anthropometric status improved in most countries, height-for-age and weight-for-age of children remained very low in 2011 in some countries including Timor-Leste, with about half of children being moderately or severely stunted. Why childhood malnutrition continues to be a persistent problem in Timor-Leste needs to be investigated so that appropriate strategies can be implemented.

The identified topics are likely to be inter-related with maternal problems leading to childhood disorders and nutritional status affected by perinatal conditions and infectious diseases. There is a clear need for more work in these areas as we identified only 28 studies that focused on safe motherhood, child health or nutrition, the majority of which were unpublished. Access to and use of health services is likely to be a very important factor underlying the identified topics. Several reasons for under-utilization of health services have been described but trends in health care utilization patterns are difficult to assess because of the non-standardized indicators from the different surveys. Community Health Integrated Service mobile clinics, which were set-up to address some of the obstacles, are being extended and may contribute to improving access to and utilization of health care. Follow-up studies to assess the impact of this and other interventions are needed.

We found that 19% of articles were on non-communicable diseases (eye and mental health), approaching the percentages for all communicable diseases (26%) and safe motherhood, child health and nutrition (25%). The relatively high number of articles on non-communicable diseases is striking considering the burden of disease information from the HMIS, priority areas of government and donor services and the Millennium Development Goals. This likely reflects the specialties of international researchers and suggests that with good support and interest, more studies and publications on the priority health needs of the Timorese is feasible.

There are several limitations of our review. Firstly, although we did an extensive search of documents from various sources, perhaps some reports and articles may have been missed. It is also possible that there are existing datasets from previously conducted research that have not be analyzed and reported. Secondly, the findings are very general; a more detailed examination of specific topics is beyond the scope of this review. Thirdly, the trends across surveys and routinely collected data should be interpreted with caution because of variations in sampling and methodology. However, in the absence of other sources of information, this is the best available method to assess gaps in knowledge and areas where more information is needed. Data from various sources were triangulated to assure validity of the main findings.

What types of future research could potentially address these priority areas? It is crucial to determine how to convert the sequence of chronically undernourished children becoming vulnerable mothers with unsafe pregnancies producing unhealthy children into a cycle of well-nourished children becoming strong mothers with safe pregnancies producing healthy children. A longitudinal cohort study conducted in a well-characterized community may provide information on how to break this intergenerational cycle of poor health. The study could follow a cohort of young women through pregnancy, evaluating them and their children in a longitudinal prospective fashion, documenting pregnancy outcomes, nutritional parameters and common infectious diseases. A cohort study such as this could yield important detailed information on the key health problems of Timorese mothers and children but may not provide an immediate and sufficient sample size to understand the causes and cause-specific burden of key morbidities. It would also require considerable resources and infrastructure. In the meanwhile, surveillance research in sentinel health centers may yield useful results on the childhood burden and causes of respiratory, febrile and diarrheal diseases, keeping in mind that a study of patients presenting for treatment will not yield information on those who remain at home. Operational research and small, less expensive descriptive or cross-sectional studies and audits in communities may also be considered to provide more information around these topics.

How can this research be carried out? There is a current lack of research infrastructure and skilled researchers in Timor-Leste. Ideally, collaborative projects with international researchers should focus on enhancing local capacity and technical expertise. Training in basic epidemiologic skills is crucial but capacity would best be built through actual conduct of research. Support for data management, analysis and reporting is also needed. Perhaps donor funding for service provision and capacity building could include a percentage dedicated to operational research.

## Conclusions

Nutrition, maternal health, childhood morbidity and mortality and access to and use of health services are recommended as priority areas for research. We think it appropriate that future research agendas in Timor-Leste focus on children and their mothers because the greatest disease burden still occurs in the youngest age groups, childhood interventions tend to be the most cost-effective and sustainable, and the development of children determines the quality of future populations. These recommendations are based on data from the last decade and will need to be updated as the population of Timor-Leste changes and develops.

## Abbreviations

CHR&D: Cabinet for Health Research and Development; HMIS: Health Management Information System.

## Competing interests

The authors declare that they have no competing interests.

## Authors’ contributions

JD, LCM, BT, JS, SL, VS, SM and KM contributed to the conception and design of the study. JD, LCM, BT, JS, SL, VS, SM, JT and KM participated in the collection and review of data sources. JD, BT and JYS analyzed the data. JD, LCM, BT, JYS, JS, SL, VS, AIS, VJ, SM, JT, JM and KM contributed to the interpretation of the results, drafting of the paper and revising it critically for substantial intellectual content. All authors read and approved the final manuscript.

## Supplementary Material

Additional file 1 List of 114 references included in the review. Click here for file
