# Supplementary material for: Identifying national health research priorities in Timor-Leste through a scoping review of existing health data
Source: Health Res Policy Syst. 2013 Mar 1;11:8. doi: 10.1186/1478-4505-11-8 (PMC3599283; doi:10.1186/1478-4505-11-8)
Supplement: Additional file 1 — List of 114 references included in the review. [file 1478-4505-11-8-S1.docx]

**List of 114 references included in the review:**

1. Agho KE, Dibley MJ, D'Este C, Gibberd R. 2008. Factors associated with haemoglobin concentration among Timor-Leste children aged 6-59 months. *J Health Popul Nutr,* **26,** 200-9.
2. Agus N, Kinyanjui V, Exposto C. 2004. Rapid Household Livelihood Security Assessment CARE International and USAID.
3. Alvaro A, Ruairí B. 2006. Rehabilitating the health system after conflict in East Timor: a shift from NGO to government leadership. *Health Policy & Planning,* **21,** 206-216.
4. Andrianopoulos T, Shanahan H. 2004. Evaluation of Mother Support Group Program. Alola Foundation and UNFPA.
5. Arcenas A, Bojo J, Larsen B, Nunez FR. 2010. The Economic Costs of Indoor Air Pollution: New Results for Indonesia, the Philippines, and Timor-Leste. *Journal of Natural Resources Policy Research,* **2,** 75-93.
6. Armstrong PK, Anstey NM, Kelly PM, Currie BJ, Martins N, Dasari P, et al. 2005. Seroprevalence of B pseudomallei. *Southeast Asian J Trop Med Public Health,* **36,** 1496-502.
7. Auliff A, Wilson DW, Russell B, Gao Q, Chen N, L.N. A, et al. 2006. Amino acid mutations in Plasmodium vivax DHFR and DHPS from several geographical regions and susceptibility to antifolate drugs. *Am J Trop Med Hyg,* **75,** 617-21.
8. Bau AM, Melito C. 2003. National Baseline Survey of Children and their Mothers. Government of Timor Leste and Germany GTZ.
9. Belton S. 2011. Health Professionals and Lawyers Understandings of Domestic Violence and the Domestic Violence Law: the 2011 Judicial System Monitoring Program survey.
10. Belton S, Whittaker A, Barclay L. 2009a. Maternal Mortality, Unplanned Pregnancy and Unsafe Abortion in Timor-Leste- A Situational Analysis.
11. Belton S, Whittaker A, Fonseca Z, Wells-Brown T, Pais P. 2009b. Attitudes towards the legal context of unsafe abortion in Timor-Leste. *Reproductive Health Matters,* **17,** 55-64.
12. Bragonier R, Nasveld P, Auliffe A. 2002a. Plasmodium malariae in East Timor. *Southeast Asian J Trop Med Public Health,* **33,** 689-90.
13. Bragonier R, Reyburn H, Nasveld P, Edstein M, Auliffe A. 2002b. Rainy-season prevalence of malaria in Bobonaro district, East Timor. *Ann Trop Med Parasitol,* **96,** 739-43.
14. Brian G, Palagyi A, Ramke J, du Toit R, Naduvilath T. 2006. Cataract and its surgery in Timor-Leste. *Clinical & experimental ophthalmology,* **34,** 870-9.
15. Brooks R, Silove D, Steel Z, Steel CB, Rees S. 2011. Explosive anger in postconflict Timor Leste: Interaction of socio-economic disadvantage and past human rights-related trauma. *J Affect Disord,* **131,** 268-76.
16. Bucens IK, Maclennan C. 2006. Survey of childhood malnutrition at Dili National Hospital, East Timor. *J Paediatr Child Health,* **42,** 28-32.
17. Bucens I, Barreto A. 2011. A three year retrospective review of Neonatal and Paediatric Inpatient Morbidity and Mortality data at the Hospital National Guido Valadares. Dili: Hospital National Guido Valadares.
18. Burns M, Baker J, Auliff AM, Gatton ML, Edstein MD, Cheng Q. 2006. Efficacy of Sulfadoxine-Pyrimethamine in the treatment of uncomplicated Plasmodium Falciparum Malaria in East Timor. *Am J Trop Med Hyg,* **74,** 361-6.
19. Cammack M, Heaton TB. 2001. Regional variation in acceptance of Indonesia's family planning program. *Population Research and Policy Review,* **20,** 565-85.
20. Chen N, J. B, N. E, Burns M, Edstein MD, Cheng Q. 2002. Short report - molecular evaluation of the efficacy of chloroquine treatment of uncomplicated plasmodium falciparum in East Timor. *Am J Trop Med Hyg,* **67,** 64-6.
21. Chevalier B, Carmoi T, Sagui E, Pierre C. 2001. Prevalence of infection with human immunodeficiency virus in East Timor. *Clin Infect Dis,* **32,** 991-2.
22. Collins SW, Martins X, Mitchell A, Teshome A, Arnason JT. 2007. Fataluku medicinal ethnobotany and the East Timorese military resistance. *Journal of ethnobiology and ethnomedicine,* **3,** 5.
23. Cooper RD, Edstein MD, Frances SP, Beebe NW. 2010. Malaria vectors of Timor-Leste. *Malaria Journal,* **9,** 40.
24. da Silva V. 2011. Knowledge, attitude and practices towards tuberculosis and socio-ecomic impact of the disease in Timor-Leste, 2010. Dili: Cabinet of Health Research and Development.
25. de Almeida A, Arez AP, Cravo PV, do Rosario VE. 2009. Analysis of genetic mutations associated with anti-malarial drug resistance in Plasmodium falciparum from the Democratic Republic of East Timor. *Malaria Journal,* **8,** 59.
26. de Almeida A, Rosario VE, Henriques G, Arez AP, Cravo P. 2010a. *Plasmodium vivax* in the Democratic Republic of East Timor: Parasite prevalence and antifolate resistance-associated mutations. *Acta tropica,* **115,** 288-92.
27. de Almeida, Rosário VED, Arez AP, Cravo P. 2010b. Malaria epidemiology in the Democratic Republic of East Timor. *Asian Pacific Journal of Tropical Medicine,* **3,** 283-287.
28. Dethlefs RF. Outcomes of cataract surgery in Timor-Leste 2010. *Clinical & experimental ophthalmology,* **40**, 332-3.
29. Dibley MJ, Senarath U, Agho KE. 2010. Infant and young child feeding indicators across nine East and Southeast Asian countries: an analysis of National Survey Data 2000?2005. *Public Health Nutrition,* **13,** 1296-1303.
30. dos Santos MML, Amaral S, Harmen SP, Joseph HM, Fernandes JL, Counahan ML. 2010. The prevalence of common skin infections in four districts in Timor-Leste: a cross sectional survey. *BMC Infectious Diseases,* **10,** 61-66.
31. du Toit R, Palagyi A, Ramke J, Brian G, Lamoureux EL. 2008. Development and validation of a vision-specific quality-of-life questionnaire for Timor-Leste. *Invest Ophthalmol Vis Sci,* **49,** 4284-9.
32. du Toit R, Palagyi A, Ramke J, Brian G, Lamoureux EL. 2010. The impact of reduced distance and near vision on the quality of life of adults in Timor-Leste. *Ophthalmology,* **117,** 2308-14.
33. Dubray C, Rose AMC. 2004. Assessment of nutritional status and vaccine coverage in Timor-Leste: Liquiça, Covalima and Bobonaro districts. Epicentre and CARE International.
34. Earnest J, Finger RP. 2009. General health in Timor-Leste: self-assessed health in a large household survey. *Aust N Z J Public Health,* **33,** 378-83.
35. Edmonds A, de Jesus RP, Lindelow M, I. M. 2005. Health Service Delivery and Utilization in Timor-Leste- A Qualitative Study. World Bank.
36. Elston JW, Bannan CL, D.T. C, Boutlis CS. 2008. Acinetobacter spp in gunshot injuries. *Emerg Infect Dis,* **14,** 178-80.
37. Ezard N, Burns M, Lynch C, Cheng Q, Edstein M. 2003. Efficacy of chloroquine in the treatment of uncomplicated Plasmodium falciparum infection in East Timor, 2000. *Acta tropica,* **88,** 87-90.
38. Finlayson J, Ghassemifar R, Holmes P, Grey D, Figliomeni L, Newbound C, et al. 2010. Hb East Timor, a variant hemoglobin associated with normal hematology. *Hemoglobin*. **34**, 561-4.
39. Guest GD, Soldanha S, Walbheim T. 2005. Back to basics: managing gunshot injuries in East Timor. *ANZ J Surg,* **75,** 220-4.
40. Harrison M. 2009. An examination of the determinants of skilled birth attendance and postpartum care in Timor-Leste: University of Washington.
41. Harrison M, Mercers MA. 2008. Maternal and Child Health in Timor-Leste: Final Knowledge, Practice and Coverage Survey Report. USAID, HAI and Ministry of Health.
42. Health Alliance International 2004a. Strengthening maternal and newborn care In Timor-Leste: Focus group discussions with midwives in Aileu, Ermera, Liquisa and Manatuto Districts. HAI and USAID.
43. Health Alliance International 2004b. Strengthening maternal and newborn care In Timor-Leste: Health facilities assessment in Aileu, Ermera, Liquisa and Manatuto Districts. HAI and USAID.
44. Health Alliance International 2005. Strengthening maternal and newborn care In Timor-Leste: Qualitative Community Assessment in Aileu and Manatuto districts. HAI and USAID.
45. Health Alliance International 2006. Increasing community demand for child spacing In Timor-Leste: Child Spacing Qualitative Community Baseline Assessment: Aileu and Manatuto Districts. HAI and USAID.
46. Health Alliance International 2009. Perinatal/infant mortality In Timor-Leste: A review of reported deaths in Health Alliance International program districts. HAI and USAID.
47. Higuchi M, Okumura J, Aoyama A, Suryawati S, Porter J. 2011. Application of standard treatment guidelines in rural community health centres, Timor-Leste. *Health policy and planning*.
48. Hynes M, Ward J, Robertson K, Crouse C. 2004. A Determination of the Prevalence of Gender-based Violence among Conflict-affected Populations in East Timor. *Disasters,* **28,** 294-321.
49. Ito M, Takasaki T, Kotaki A, Tajima S, Yuwono D, H.S. R, et al. 2010. Molecular and virological analyses of dengue virus responsible for dengue outbreak in East Timor in 2005. *Jpn J Infect Dis,* **63,** 181-4.
50. Kalayanarooj S, Rimal HS, Andjaparidze A, Vatcharasaevee V, Nisalak A, Jarman RG, et al. 2007. Clinical intervention and molecular characteristics of a dengue hemorrhagic fever outbreak in Timor Leste, 2005. *Am J Trop Med Hyg,* **77,** 534-7.
51. Kelly PM, Lumb R, Pinto A, da Costa G, Sarmento J, Bastian I. 2005. Analysis of *Mycobacterium tuberculosis* isolates from treatment failure patients living in East Timor. *Int J Tuberc Lung Dis,* 9**,** 81-6.
52. Kennedy E, Gray N, Azzopardi P, Creati M. 2011. Adolescent fertility and family planning in East Asia and the Pacific: a review of DHS reports. *Reproductive health*, **8**, 11.
53. Knight F. Pathways to Safer Motherhood in Bobonaro District, Timor-Leste: University of Adelaide; 2009.
54. Lee J, Rawstorne P, Worth H. 2009. Behavioural surveillance survey In Timor‐Leste: First round results for female sex workers: University of New South Wales.
55. Lee J, Rawstorne P, Worth H. 2009. Behavioural surveillance survey in Timor‐Leste: First round results for uniformed personnel: University of New South Wales.
56. Livermore C. 2002. *Tuur Ahi:* Childbirth and child death in Aileu, East Timor.
57. Loughry M, Kostelny K. 2002. Mapping Psychosocial Interventions in East Timor. Refugee Studies Centre, University of Oxford.
58. Lover AA, Sutton BA, Asy AJ, Wilder-Smith A. 2011. An exploratory study of treated-bed nets in Timor-Leste: patterns of intended and alternative usage. *Malaria Journal,* **10**, 199.
59. Marlowe P, Mahmood MA. 2009. Public health and health services development in postconflict communities: a case study of a safe motherhood project in East Timor. *Asia Pac J Public Health,* **21,** 469-76.
60. Martins J, Zwi A, Kelly P. 2010. What has been the contribution of the first Global Fund grant (2003-2006) to malaria control and health system strengthening in Timor-Leste? *Malaria Journal,* **9,** 1-2.
61. Martins JS, Zwi AB, Martins N, Kelly PM. 2009a. Malaria control in Timor-Leste during a period of political instability: what lessons can be learned? *Confl Health,* **3,** 11.
62. Martins N, Heldal E, Sarmento J, Araujo RM, Rolandsen EB, Kelly PM. 2006. Tuberculosis control in conflict-affected East Timor, 1996–2004. *Int J Tuberc Lung Dis*, **10**, 975–81
63. Martins N, Grace J, Kelly PM. An ethnographic study of barriers to and enabling factors for tuberculosis treatment adherence in Timor-Leste. Int J Tuberc Lung Dis. 2008; 12(5): 532-7.
64. Martins N, Kelly PM, Grace JA, Zwi AB. 2006. Reconstructing Tuberculosis Services after Major Conflict- Experiences and Lessons Learned in East Timor. *PLoS Medicine,* **3,** e383.
65. Martins N, Morris P, Kelly PM. 2009b. Food incentives to improve completion of tuberculosis treatment: randomised controlled trial in Dili, Timor-Leste. *BMJ,* **339,** b4248-b4248.
66. McAuliffe AV, Grootjans J, Fisher JEM. 2002. Hasten slowly: a needs analysis for nurse and village health worker education in East Timor. *International Nursing Review,* 49**,** 47-53.
67. Ministry of Health of Timor-Leste. 2004. EPI coverage field cluster surveys in the Democratic Republic of Timor Leste,
68. Ministry of Health of Timor-Leste. 2008. Hospital Costing Study in the Democratic Republic of Timor-Leste.
69. Moore MH, Fernandes AL. 2006. Cleft surgery in East timor: the first four years. *ANZ J Surg,* **76,** 683-7.
70. Neto F, Furnham A, Pinto Mda C. 2009. Estimating one's own and one's relatives' multiple intelligence: a cross-cultural study from East Timor and Portugal. *Span J Psychol,* 12**,** 518-27.
71. OXFAM. 2007 Timor-Leste Food Security Baseline Survey Report: OXFAM Australia.
72. Palagyi A, Brian G, Ramke J. 2010. Training and using mid‐level eye care workers: early lessons from Timor‐Leste. *Clinical and Experimental Ophthalmology (formerly Australian and New Zealand Journal of Ophthalmology),* **38,** 805-811.
73. Palagyi A, Ramke J, du Toit R, Brian G. 2008. Eye care in Timor-Leste: a population-based study of utilization and barriers. *Clinical & experimental ophthalmology,* 36**,** 47-53.
74. Pisani E. 2004. HIV, STIs and risk behavior in East Timor an historic opportunity for effective action. USAID and Family Health International.
75. Pisani E, Purnomo H, Sutrisna A, Asy A, Zaw M, Tilman C, et al. 2006. Basing policy on evidence: low HIV, STIs, and risk behaviour in Dili, East Timor argue for more focused interventions. *Sex Transm Infect,* 82**,** 88-93.
76. Ramke J. 2011. Vision screening of children attending primary school in rural Timor-Leste. *Clinical and Experimental Ophthalmology (formerly Australian and New Zealand Journal of Ophthalmology)***,** **39**, 377-8.
77. Ramke J, Brian G. 2009. Letter to the Editor: Are readymade spectacles sufficient in developing countries? *Clinical & experimental ophthalmology,* **37,** 900-2.
78. Ramke J, du Toit R, Palagyi A, Brian G, Naduvilath T. 2007a. Correction of refractive error and presbyopia in Timor-Leste. *Br J Ophthalmol,* **91,** 860-6.
79. Ramke J, Palagyi A, du Toit R, Brian G. 2009a. Applying Standards to Readymade Spectacles Used in Low-Resource Countries. *Optometry and Vision Science,* **86,** 1104-11.
80. Ramke J, Palagyi A, du Toit R, Brian G. 2009b. Stated and actual willingness to pay for spectacles in Timor-Leste. *Ophthalmic epidemiology,* **16,** 224-30.
81. Ramke J, Palagyi A, Naduvilath T, du Toit R, Brian G. 2007b. Prevalence and causes of blindness and low vision in Timor-Leste. *The British journal of ophthalmology,* **91,** 1117-21.
82. Ramke J, Qoqonokana MQ, Brian G. 2011. Letter to the Editor: Diabetes and its ocular complications in Timor-Leste. *Clinical & experimental ophthalmology*, **39**, 843-4.
83. Richter K. 2009. Changes in Subjective Well-Being in Timor-Leste on the Path to Independence. *World Development,* **37,** 371-384.
84. Rogers A. 2001. Treating Child Illness in Dili: A Household Level Analysis. Yale University.
85. Saikia U, Hosgelen M. 2010. Timor-Leste’s demographic destiny and its implications for the health sector by 2020. *Journal of Population Research,* **27,** 133-146.
86. Santos C, Phillips C, Fondevila M, Porras-Hurtado L, Carracedo A, Souto L, et al. 2011. A study of East Timor variability using the SNPforID 52-plex SNP panel. *Forensic science international. Genetics,* **5,** e25-6.
87. Senarath U, Dibley MJ, Agho KE. 2007. Breastfeeding practices and associated factors among children under 24 months of age in Timor-Leste. *Eur J Clin Nutr,* **61,** 387-97.
88. Senarath U, Dibley MJ, Agho KE. 2010. Factors associated with nonexclusive breastfeeding in 5 east and southeast Asian countries: a multilevel analysis. *J Hum Lact,* **26,** 248-57.
89. Shiokawa H. 2010. Children draw with rulers: a symbolic sign of post-conflict adjustment? *Pediatrics International,* **52,** 327-328.
90. Silove D, Bateman CR, Brooks RT, Fonseca CAZ, Steel Z, Rodger J, et al. Estimating Clinically Relevant Mental Disorders in a Rural and an Urban Setting in Postconflict Timor-Leste. Archives of General Psychiatry. 2008; 65(10): 1205-12.
91. Silove D, Brooks R, Bateman CS, Steel Z, Amaral ZFC, Rodger J, et al. 2010. Social and trauma related pathways leading to psychological distress and functional limitations four years after the humanitarian emergency in Timor Leste. *Journal of Traumatic Stress,* 23**,** 151-160.
92. Silove D, Brooks R, Bateman Steel CR, Steel Z, Hewage K, Rodger J, et al. 2009. Explosive anger as a response to human rights violations in post-conflict Timor-Leste. *Soc Sci Med*, **69**, 670-7
93. Silove D, Manicavasagar V, Baker K, Mausiri M, Soares M, de Carvalho F, et al2004. Indices of social risk among first attenders of an emergency mental health service in post-conflict East Timor: an exploratory investigation. *Australian and New Zealand Journal of Psychiatry,* **38,** 929-32.
94. Sinha DN, Palipudi KM, Rolle I, Asma S, Rinchen S. 2011. Tobacco use among youth and adults in member countries of South-East Asia region: review of findings from surveys under the Global Tobacco Surveillance System. *Indian J Public Health,* **55,** 169-76.
95. Siziya S, Muula AS, Rudatsikira E. 2008. Prevalence and correlates of current cigarette smoking among adolescents in East Timor-Leste. *Indian Pediatr,* **45,** 963-8.
96. Soares AMRPB, Martins J, Martins N, de Jesus RP, Gueteres A, Grace J, et al. 2004. The Knowledge, Attitude and Practice on Malaria in Timor-Leste. Universidade da Paz, Dili, Timor-Leste and Menzies School of Health Research, Darwin, NT, Australia.
97. Somerville E, Somerville H, Soares A, Silove D. Cultural Attitudes and Beliefs concerning Epilepsy in East Timor. 26th International Epilepsy Congress, 2005 Paris, France. *Epilepsia*, 351.
98. Souto L, Alves C, Gusmao L, Ferreira E, Amorim A, Corte-Real F, et al. 2005. Population data on 15 autosomal STRs in a sample from East Timor. *Forensic science international,* **155,** 77-80.
99. Souto L, Gusmao L, Ferreira E, Amorim A, Corte-Real F, Vieira DN. 2006. Y-chromosome STR haplotypes in East Timor: forensic evaluation and population data. *Forensic science international,* 156**,** 261-5.
100. Sullivan KH, Gillies R, Zwi A. 2009. Towards a trauma-less Timor-Leste: An exploration of hospital-based trauma care in Timor-Leste using the ‘WHO-IATSC’ Essential Trauma Care guidelines. *ANZ Journal of Surgery*, **79** (Suppl 1): A87.
101. Timor-Leste Asistensia Integradu Saude. 2007. Community consultation on child health practices in Timor-Leste, TAIS and USAID.
102. Timor-Leste Asistensia Integradu Saude. 2009. Evaluation of National Family Health Promoter Program. TAIS and USAID.
103. Thakur JS, Garg R, Narain JP, Menabde N. 2011. Tobacco use: a major risk factor for non communicable diseases in South-East Asia region. *Indian J Public Health,* **55,** 155-60.
104. Upul S, Michael JD, Kingsley EA. 2007. Breast-feeding Performance Index: a composite index to describe overall breast-feeding performance among infants under 6 months of age. *Public Health Nutrition,* **10,** 996-1004.
105. van Schoor V. 2003. Maternal Health - Home Births and Post-Partum Care in East Timor: a community-based qualitative study.
106. Wayte K, Barclay L, Kelly P. 2007. Improving Access to Care: Birth Facilities and Maternity Waiting Homes in Timor-Leste. Menzies school of Health Research; Charles Darwin University.
107. Wayte K, Zwi A, Belton S, Martins J, Martins N, Whelan A, et al. 2008. Conflict and Development: Challenges in Responding to Sexual and Reproductive Health Needs in Timor-Leste. *Reproductive Health Matters,* **16,** 83-92.
108. Wild K, Barclay L, Kelly P, Martins N. 2010. Birth choices in Timor-Leste: a framework for understanding the use of maternal health services in low resource settings. *Soc Sci Med,* **71,** 2038-45.
109. Wild K, Kelly P, Martins J, Barclay L. 2007. Culture beliefs and birth: An exploration of birthing practices in Timor-Leste.
110. World Health Organization. 2005. Dengue haemorrhagic fever, Timor-Leste--Update. *Wkly Epidemiol Rec*, **80**, 85-6.
111. World Health Organization. 2005. Dengue haemorrhagic fever, Timor-Leste. *Wkly Epidemiol Rec*, **80**, 61.
112. World Health Organization. 2001. Final report on outbreak of diarrhoea in Oecussi Distict, East Timor.
113. Zwi AB, Blignault I, Glazebrook D, Correia V, Bateman Steel CR, Ferreira E, Pinto BM. 2009. Timor‐Leste health care seeking behaviour study. The University of New South Wales, Sydney.
114. Zwi AB, Martins J, Grove NJ, Wayte K, Martins N, Kelly P, et al. 2007. Health sector resilience and performance in a time of instability. The University of New South Wales, Sydney.
